# Supplementary material for: Orai inhibition modulates pulmonary ILC2 metabolism and alleviates airway hyperreactivity in murine and humanized models
Source: Nat Commun. 2023 Sep 26;14:5989. doi: 10.1038/s41467-023-41065-4 (PMC10522697; doi:10.1038/s41467-023-41065-4)
Supplement: Supplementary file 1 — Supplementary Information [file 41467_2023_41065_MOESM1_ESM.pdf]

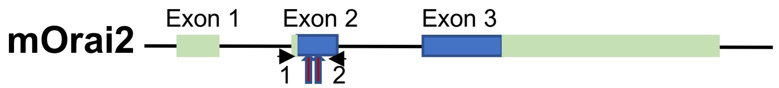


```
WT   gcacaagaactatctaagatgctccaacgtgtcacgtctcttcctccccagCCTGGTCCC  60
KO   GCACAAGAACTATCTAAGATGCTCCAACGTGTCACGTCTCTTCCTCCCCAGCCTGGTCCC  60
     ************************************************************

WT   CACCATGAGTGCAGAGCTCAATGTGCCCATGGACCCCTCTGCCCCTGCCTGCCCTGAACC  120
KO   CACCATGAGTGCAGAGCTCAATGTGCCCATGGACCCCTCTGCCCC---------------  105
     *********************************************

WT   CGGCCACAAGGGCATGGATTACCGAGACTGGGTCCGGCGCAGCTACCTGGAACTCGTCAC  180
KO   ----------------------------TGGGTCCGGCGCAGCTACCTGGAACTCGTCAC  137
                                 ********************************

WT   GTCTAACCACCATTCGGTGCAG       202
KO   GTCTAACCACCATTCGGTGC--       157
     ********************
```

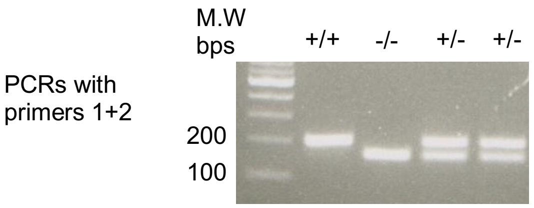

Figure S1. Targeting strategy for murine *Orai2* gene. Mouse *Orai2* gene comprises of 3 exons, with Exon 2 encoding translational start codon. Two gRNAs targeting exon 2 (shown as arrows) were used to excise a 43 bp fragment (seen in the sequencing results below), resulting in a change in reading frame. PCR using indicated primers (1 and 2) allows for distinguishing between WT, heterozygous and knockout animals as shown at the bottom.

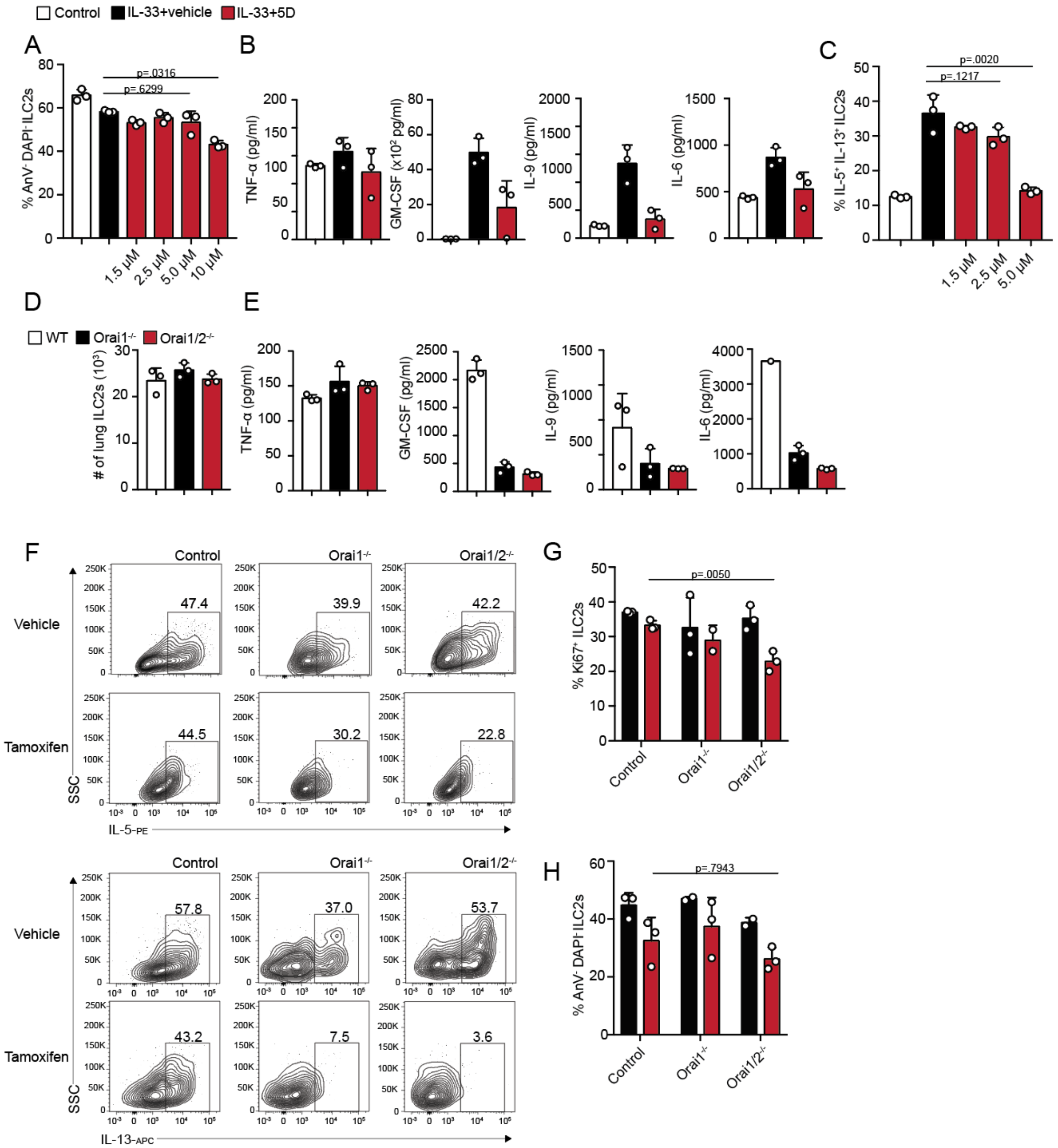

Figure S2. Pulmonary ILC2 effector function is dependent on Orai1 and Orai2. (**A**) Live ILC2s measured by Annexin V and DAPI via flow cytometry after varying doses of 5D. (**B**) Additional cytokines secreted in the supernatant of ILC2s cultured with and without 5D after 48 hours measure by Legendplex. (**C**) IL-5+ IL-13+ ILC2s after culture with varying doses of compound 5D. (**D**) Number of pulmonary ILC2s isolated from naïve wild-type (WT), *Orai1*$^{-/-}$ and *Orai1/2*$^{-/-}$ mice. (**E**) Additional cytokines secreted in the supernatant of ILC2s from indicated mice after 48 hours measure by Legendplex. (**F**) Representative flow cytometry plots demonstrating IL-5 and IL-13 production intracellularly in WT, *Orai1*$^{-/-}$ and *Orai1/2*$^{-/-}$ mice. (**G**) Proliferation of ILC2s measured by Ki67 via flow cytometry in WT, *Orai1*$^{-/-}$ and *Orai1/2*$^{-/-}$ mice. (**H**) Live ILC2s measured by Annexin V and DAPI via flow cytometry in WT, *Orai1*$^{-/-}$ and *Orai1/2*$^{-/-}$ mice. Data are representative of two independent experiments and are presented as means ± SEM. Source data are provided as a Source Data file. A two-tailed Student's *t* test for unpaired data was applied for comparisons between two groups, except for multi-group comparisons where Tukey's multiple comparison one-way ANOVA tests were used.

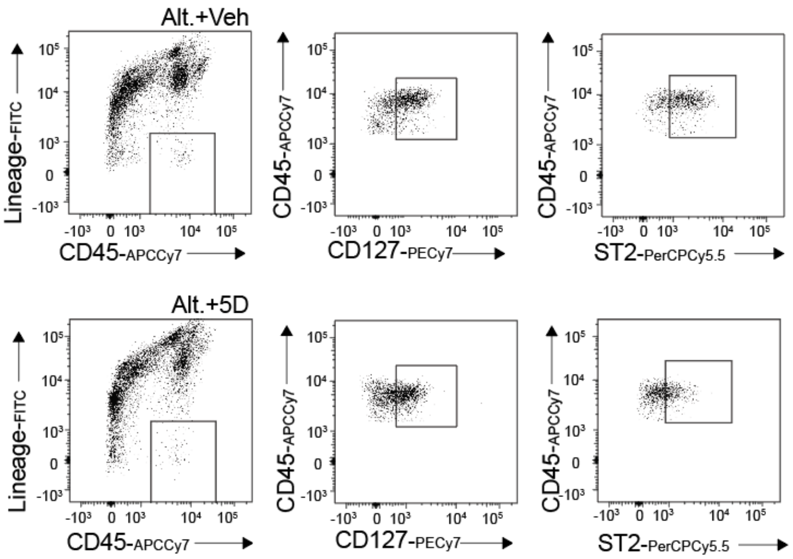

Figure S3. Inhibition of Orai channels in pulmonary ILC2s significantly downregulates development of airway inflammation. (A) Full gating strategy of ILC2s in WT mice with and without compound 5D via flow cytometry.

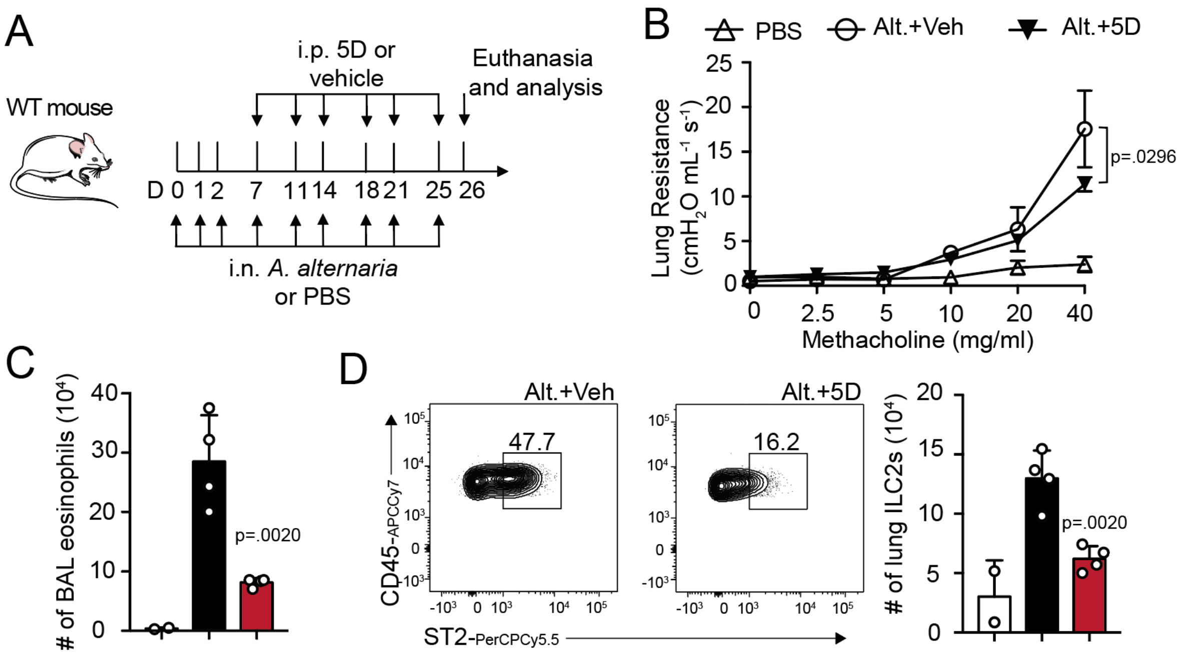

Figure S4. Inhibition of Orai channels in pulmonary ILC2s significantly downregulates development of airway inflammation. (**A**) A cohort of BALB/cBYJ mice were challenged intranasally with *Alternaria alternata* (*A. alternata*) or PBS for three consecutive days the first week of the model. They were then challenged every Monday and Friday for the next three following weeks. Mice were also given injections of 5D or vehicle. On day 26, AHR (**B**) was assessed. Additionally total number of eosinophils in the BAL (**C**), and number of lung ILC2s (**D**) is presented as mean numbers +/- SEM. *n* = 4 biologically independent mice. Data are representative of two independent experiments and are presented as means ± SEM. Source data are provided as a Source Data file. A two-tailed Student's *t* test for unpaired data was applied for comparisons between two groups, except for multi-group comparisons where Tukey's multiple comparison one-way ANOVA tests were used. Mouse image provided with permission from Servier Medical Art.
