# Supplementary material for: Orai inhibition modulates pulmonary ILC2 metabolism and alleviates airway hyperreactivity in murine and humanized models
Source: Nat Commun. 2023 Sep 26;14:5989. doi: 10.1038/s41467-023-41065-4 (PMC10522697; doi:10.1038/s41467-023-41065-4)
Supplement: Supplementary file 3 — Reporting Summary [file 41467_2023_41065_MOESM3_ESM.pdf]

nature portfolio

Corresponding author(s): Dr. Omid Akbari

Last updated by author(s): 8/10/23

Reporting Summary

Nature Portfolio wishes to improve the reproducibility of the work that we publish. This form provides structure for consistency and transparency in reporting. For further information on Nature Portfolio policies, see our Editorial Policies and the Editorial Policy Checklist.

Statistics

For all statistical analyses, confirm that the following items are present in the figure legend, table legend, main text, or Methods section.

n/a	Confirmed	
☐	☒	The exact sample size (*n*) for each experimental group/condition, given as a discrete number and unit of measurement
☐	☒	A statement on whether measurements were taken from distinct samples or whether the same sample was measured repeatedly
☐	☒	The statistical test(s) used AND whether they are one- or two-sided<br>*Only common tests should be described solely by name; describe more complex techniques in the Methods section.*
☒	☐	A description of all covariates tested
☒	☐	A description of any assumptions or corrections, such as tests of normality and adjustment for multiple comparisons
☐	☒	A full description of the statistical parameters including central tendency (e.g. means) or other basic estimates (e.g. regression coefficient) AND variation (e.g. standard deviation) or associated estimates of uncertainty (e.g. confidence intervals)
☐	☒	For null hypothesis testing, the test statistic (e.g. *F*, *t*, *r*) with confidence intervals, effect sizes, degrees of freedom and *P* value noted<br>*Give P values as exact values whenever suitable.*
☒	☐	For Bayesian analysis, information on the choice of priors and Markov chain Monte Carlo settings
☒	☐	For hierarchical and complex designs, identification of the appropriate level for tests and full reporting of outcomes
☒	☐	Estimates of effect sizes (e.g. Cohen's *d*, Pearson's *r*), indicating how they were calculated

Our web collection on statistics for biologists contains articles on many of the points above.

Software and code

Policy information about availability of computer code

Data collection	BD FACSDiva software v8.0.1 was used for flow cytometry data acquisition.
Data analysis	Partek Genomics Suite software 7.0, Flowjo software (TreeStar) version 9, GraphpadPrism software v8, Seahorse data analytics (https://seahorseanalytics.agilent.com), STAR – 2.6.1d .

For manuscripts utilizing custom algorithms or software that are central to the research but not yet described in published literature, software must be made available to editors and reviewers. We strongly encourage code deposition in a community repository (e.g. GitHub). See the Nature Portfolio guidelines for submitting code & software for further information.

Data

Policy information about availability of data

All manuscripts must include a data availability statement. This statement should provide the following information, where applicable:

- Accession codes, unique identifiers, or web links for publicly available datasets
- A description of any restrictions on data availability
- For clinical datasets or third party data, please ensure that the statement adheres to our policy

The RNA-seq data from Figure3 have been deposited in the Genbank database under the GEO accession code GSE221009, https://www.ncbi.nlm.nih.gov/geo/query/acc.cgi?acc=GSE221009.
Single-cell RNA sequencing data is found in the Genbank database under GEO accession code GSE102299, https://www.ncbi.nlm.nih.gov/geo/query/acc.cgi?acc=GSE102299. All data are included in the Supplemental Information. The raw numbers for charts and graphs are available in the Source Data file whenever possible. Source data are provided with this paper.

Human research participants

Policy information about studies involving human research participants and Sex and Gender in Research.

Reporting on sex and gender	NA
Population characteristics	NA
Recruitment	A total of 14 healthy volunteers (7 males, 7 females) with written consent participated in the study, aged 18-70, no compensation was provided.
Ethics oversight	NA

Note that full information on the approval of the study protocol must also be provided in the manuscript.

Field-specific reporting

Please select the one below that is the best fit for your research. If you are not sure, read the appropriate sections before making your selection.

☒ Life sciences ☐ Behavioural & social sciences ☐ Ecological, evolutionary & environmental sciences

For a reference copy of the document with all sections, see nature.com/documents/nr-reporting-summary-flat.pdf

Life sciences study design

All studies must disclose on these points even when the disclosure is negative.

Sample size	We used ANOVA to calculate the sample size with the assumption of power of 80%, effect size of ˜50 and alpha less than 0.05.
Data exclusions	No animals or samples were excluded. There was no anticipation of excluding animals once they underwent the
Replication	experiments. All attempts at replication were successful, experiments were repeated 2 to 3 times.
Randomization	Age-matched female mice were randomly allocated to experimental groups. Randomization process consisted of pooling all the mice in a large cage and random picking out allocating to experimental groups.
Blinding	Investigators were blinded to group allocation during data collection and analysis except for flow cytometry as the design and conditions prevent possible sources of bias.

Reporting for specific materials, systems and methods

We require information from authors about some types of materials, experimental systems and methods used in many studies. Here, indicate whether each material, system or method listed is relevant to your study. If you are not sure if a list item applies to your research, read the appropriate section before selecting a response.

Materials & experimental systems

n/a	Involved in the study	
☐	☒	Antibodies
☒	☐	Eukaryotic cell lines
☒	☐	Palaeontology and archaeology
☐	☒	Animals and other organisms
☒	☐	Clinical data
☒	☐	Dual use research of concern

Methods

n/a	Involved in the study	
☒	☐	ChIP-seq
☐	☒	Flow cytometry
☒	☐	MRI-based neuroimaging

Antibodies

Antibodies used

PECy7 anti-mouse CD45 (30-F11, #103114), APCCy7 anti-mouse CD45 (30-F11, #103116), FITC anti-mouse CD3 (17A2, #100204), APCCy7 anti-mouse CD11c (N418, #117324), PerCPCy5.5 anti-mouse CD11c (N418, #117328), APC anti-mouse CD170 (SiglecF, S17007L, #155507), FITC anti-mouse CD19 (6D5, #115506), APC anti-mouse Gr-1 (RB6-8C5, #108412), PE-Cy7 anti-mouse CD127 (A7R34, #135014), PE anti-mouse IL-5 (TRFK5, #504304), FITC anti-mouse CD5 (53-7.3, #100606), FITC anti-mouse TCR (H57-597, #109206), FITC anti-mouse CD45R (RA3-6B2, #103206), FITC anti-mouse Gr-1 (RB6-8C5, #108406), FITC anti-mouse CD11c (N418, # 117306), FITC anti-mouse CD11b (M1/70, # 101206), FITC anti-mouse Ter119 (TER-119, #116206), FITC anti-mouse Fc RI (MAR-1, #134306), FITC anti-mouse TCR (eBioGL3, #107504), FITC anti-mouse CD335 (29A1.4, #137606) all from Biolegend. PE anti-mouse CD170 (SiglecF, E50-2440, #552126) from BD Biosciences and eFluor450 anti-mouse CD11b (M1/70, #48-0112-82), PerCP-eFluor710 anti-mouse ST2 (RMST2-2, #46-9335-82) APC anti-mouse Ki67 (SolA15, #17-5698-82), APC anti-mouse IL-13 (85BRD, 17-7136-42) from Thermofisher. All antibodies listed above were used at a 1:300 dilution, except the intracellular antibodies at 1:100. Rabbit Polyclonal ORAI2 (#NBP2-76955) and rabbit polyclonal Orai1 (#NBP1-77283) were purchased from Novus Biologicals and used at a 1:100 dilution. Alexa Fluor 647 goat anti-rabbit (#111-606-047) was purchased from Jackson ImmunoResearch Laboratories and used at a 1:100 dilution.

Validation	All antibodies are from commercial source and have been validated by the vendors and their validation data are available on the manufacturers' websites. Specifically, each antibody was validated by flow cytometry using either a cell line with high specific antigen expression or with primary cells isolated from the mouse that express the relevant antigen.

Animals and other research organisms

Policy information about studies involving animals; ARRIVE guidelines recommended for reporting animal research, and Sex and Gender in Research

Laboratory animals	Five-to-eight week old female mice were used in this study. Wild type (WT) BALB/cByJ (stock #001026) and Rag2-/- gc-/-(C;129S4-Rag2tm1.1Flv Il2rgtm1.1Flv/J, stock # 014593) were purchased from the Jackson Laboratories (Ann Harbor, ME). Orai1-deficient mice have been previously reported and CRISPR/Cas9 was used to delete Orai2 gene to create Orai1/2 mice. Crossed mice were genotyped following protocol detailed in the original publication describing Orai1 mice. All mice were bred separately in specific pathogen- free conditions in the mouse facility at the Keck School of Medicine, University of Southern California (USC) and maintained at a macroenvironmental temperature of 21–21–22°C, humidity (48–52%), in a conventional 12:12 light/dark cycle with lights on at 6:00 a.m. and off at 6:00 p
Wild animals	No wild animals were used in this study
Reporting on sex	Females were used as males housed in cages engage in frequent fighting which brings bias to the immunological readout upon study completion.
Field-collected samples	No field-collected samples were used in this study.
Ethics oversight	All mice were maintained and bred in a pathogen free mouse colony at the Keck School of Medicine, University of Southern California under protocols approved by the Institutional Animal Care and Use Committee.

Note that full information on the approval of the study protocol must also be provided in the manuscript.

Flow Cytometry

Plots

Confirm that:

☒ The axis labels state the marker and fluorochrome used (e.g. CD4-FITC).

☒ The axis scales are clearly visible. Include numbers along axes only for bottom left plot of group (a 'group' is an analysis of identical markers).

☒ All plots are contour plots with outliers or pseudocolor plots.

☒ A numerical value for number of cells or percentage (with statistics) is provided.

Methodology

Sample preparation	Lungs were perfused with ice cold PBS through the left ventricle of the heart, digested with 400KU/ml Collagenase IV (Worthington) for one hour at 37 C, minced through a 70μ m cell strainer and red blood cells lysed. Single cell suspensions were then used for the selected readouts.
Instrument	FACSCanto II and/or FACSAria III
Software	FACSDiva version 8.0.1
Cell population abundance	10000-100000 ILC2S were sorted per mouse. Purity was assessed by analyzing sorted cells for the same markers used for sorting. Purity was always over 90%.
Gating strategy	ILC2s; lineage-CD45+CD127+ST2+

☒ Tick this box to confirm that a figure exemplifying the gating strategy is provided in the Supplementary Information.
